# Synergistic Alleviation of Saline–Alkali Stress and Enhancement of Selenium Nutrition in Rice by ACC (1-Aminocyclopropane-1-Carboxylate) Deaminase-Producing *Serratia liquefaciens* and Biogenically Synthesized Nano-Selenium

**DOI:** 10.3390/plants14152376

**Published:** 2025-08-01

**Authors:** Nina Zhu, Xinpei Wei, Xingye Pan, Benkang Xie, Shuquan Xin, Kai Song

**Affiliations:** 1 School of Life Science, Changchun Normal University, Changchun 130032, China; znn0431@163.com (N.Z.); weixinpei@yeah.net (X.W.); pxy_respect@163.com (X.P.); kkangyyds@163.com (B.X.); 2 Institute of Innovation Science and Technology, Changchun Normal University, Changchun 130032, China

**Keywords:** *Serratia liquefaciens*, ACC deaminase, nano-selenium, rice, rhizosphere microbiota

## Abstract

Soil salinization and selenium (Se) deficiency threaten global food security. This study developed a composite bioinoculant combining ACC deaminase-producing *Serratia liquefaciens* and biogenically synthesized nano-selenium (SeNPs) to alleviate saline–alkali stress and enhance Se nutrition in rice (*Oryza sativa* L.). A strain of *S. liquefaciens* with high ACC deaminase activity was isolated and used to biosynthesize SeNPs with stable physicochemical properties. Pot experiments showed that application of the composite inoculant (S3: *S. liquefaciens* + 40 mmol/L SeNPs) significantly improved seedling biomass (fresh weight +53.8%, dry weight +60.6%), plant height (+31.6%), and root activity under saline–alkali conditions. S3 treatment also enhanced panicle weight, seed-setting rate, and grain Se content (234.13 μg/kg), meeting national Se-enriched rice standards. Moreover, it increased rhizosphere soil N, P, and K availability and improved microbial α-diversity. This is the first comprehensive demonstration that a synergistic bioformulation of ACC deaminase PGPR and biogenic SeNPs effectively mitigates saline–alkali stress, enhances soil fertility, and enables safe Se biofortification in rice.

## 1. Introduction

Microbial biofertilizers are environmentally friendly alternatives to chemical fertilizers, capable of enhancing crop yields with greater cost-effectiveness [1]. Soil salinization is an increasingly severe threat to global food production, adversely affecting plant growth through osmotic stress, ionic toxicity, and oxidative damage [2,3,4]. One of the key physiological responses of plants under salt stress is the overaccumulation of ethylene, which suppresses root elongation and exacerbates growth inhibition [2,3,4]. Ethylene homeostasis in plants can be modulated by microbial intervention. Plant growth-promoting rhizobacteria (PGPR) that produce 1-aminocyclopropane-1-carboxylate (ACC) deaminase can hydrolyze ACC, the direct precursor of ethylene, into α-ketobutyrate and ammonia, thereby mitigating ethylene-induced growth suppression [5,6,7]. Studies have shown that high salinity impairs rice physiology, biochemical pathways [8], and gene expression, whereas PGPR with ACC deaminase activity enhances stress tolerance in crops such as rice and tomato through hormonal regulation and rhizosphere colonization [9,10].

Concurrently, selenium (Se) is an essential micronutrient for humans and animals; however, its bioavailability in agricultural soils is generally low [11,12]. Inorganic selenium forms (Se^4+^/Se^6+^) pose toxicity risks and have low bioavailability in plants [13,14]. Nano-selenium (SeNPs), especially those biosynthesized by microbes via the reduction of selenite, exhibit lower toxicity, higher biological activity, and improved bioavailability compared to traditional Se compounds [15,16]. Due to the moderate stability of selenium compounds, excess Se can disrupt biological functions; however, reductive organosulfur compounds such as methionine can alleviate Se toxicity. Several bacterial strains are known to produce selenocysteine, which is converted into selenomethionine via the methionine synthesis pathway [17,18].

The synergistic interaction between nanoparticles and PGPR has been reported to enhance antioxidant enzyme activity, solute accumulation, plant health, and productivity under various environmental stresses, including salinity, heat, drought, and heavy metal exposure [19]. However, the joint application of PGPR and SeNPs for concurrent abiotic stress mitigation and nutritional enhancement remains underexplored—particularly under saline–alkali stress conditions.

Soil microbiota play a pivotal role in plant development and nutrient acquisition by decomposing organic matter, forming humus, and participating in nutrient cycling [6]. Microbial community structures are highly sensitive to environmental factors such as vegetation type, soil composition, tillage, and fertilization [20]. PGPR not only improve plant adaptability and mitigate environmental stress but also contribute to nutrient mobilization by solubilizing minerals and fixing atmospheric nitrogen [21,22,23].

Rice (*Oryza sativa* L.) is notably more sensitive to salinity in the rhizosphere compared to other cereals [8]. Based on this, we hypothesized that a composite formulation combining ACC deaminase-producing bacteria and biogenic SeNPs could synergistically alleviate saline–alkali stress in rice, improve soil nutrient status, and enhance Se biofortification. To test this hypothesis, the present study was designed to (1) isolate and identify a high-efficiency ACC deaminase-producing PGPR strain from rhizosphere soil; (2) biosynthesize and characterize nano-selenium using the isolated strain; (3) develop a composite bioinoculant; and (4) evaluate its effects on rice growth, Se accumulation, rhizosphere soil nutrient profiles, and microbial community structure under controlled saline–alkali stress conditions.

## 2. Results

### 2.1. Screening and Identification of ACC Deaminase-Producing Strain and Biosynthesis of Nano-Selenium

**Figure 1:**
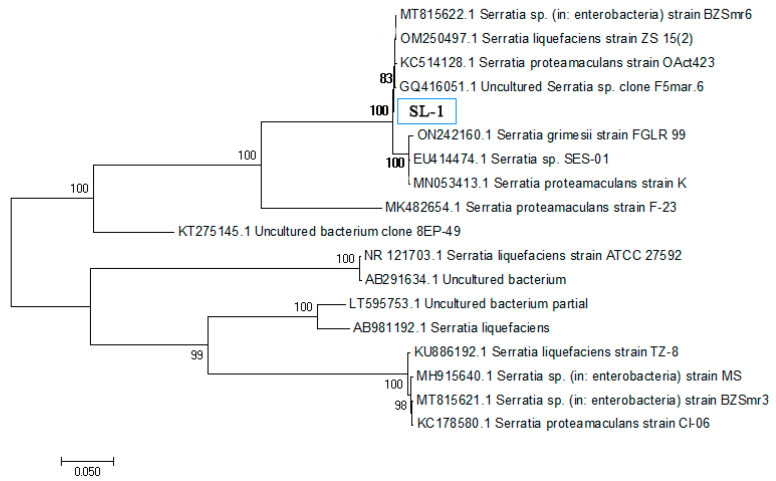
Phylogenetic tree of strain SL-1 and closely related species based on 16S rRNA gene sequences. Constructed using the neighbor-joining method. GenBank accession numbers are shown in parentheses. The scale bar indicates nucleotide substitution rate.

A strain exhibiting significant ACC deaminase activity was isolated from rice rhizosphere soil using selective medium with ACC as the sole nitrogen source and designated SL-1. The specific ACC deaminase activity of strain SL-1 reached 0.084 U/mg protein, ranking highest among all candidate strains. A 16S rRNA gene sequencing revealed 99.5% similarity between SL-1 and *Serratia liquefaciens*. Phylogenetic analysis confirmed its taxonomic position within the phylum Proteobacteria, clustering closely with previously reported Serratia strains with known plant growth-promoting and stress tolerance traits (Figure 1).

**Figure 2:**
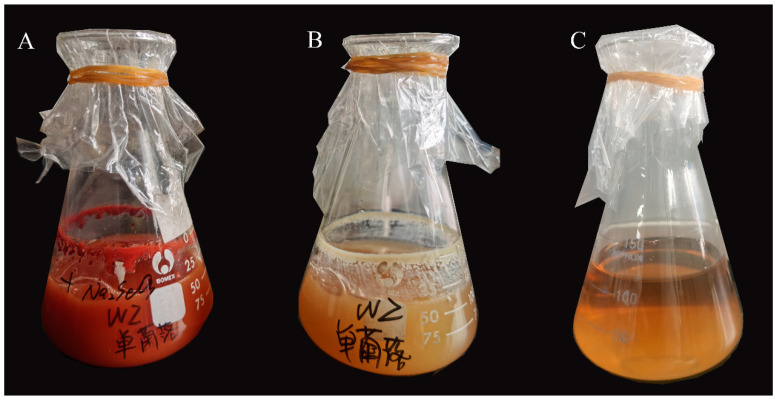
Color change of LB culture media during SeNPs biosynthesis by *S. liquefaciens* SL-1. (**A**) SL-1 + Na_2_SeO_3_ (5 mmol/L); (**B**) SL-1 without Na_2_SeO_3_; (**C**) Na_2_SeO_3_ without SL-1 inoculation.

Upon inoculation of activated *S. liquefaciens* SL-1 into LB broth supplemented with 5 mmol/L Na_2_SeO_3_ and incubation for 48 h, the culture broth changed from pale yellow to brick red (Figure 2), indicating successful bioreduction of sodium selenite to elemental selenium nanoparticles (SeNPs).

To evaluate the salinity tolerance of strain SL-1 and its implications for application under saline–alkali stress, the bacterium was cultured in LB medium amended with NaCl/Na_2_CO_3_ (1:1 ratio) at concentrations ranging from 0 to 500 mM, corresponding to electrical conductivity (EC) levels up to approximately 5.6 dS/m as used in the pot experiments. After 24 h of incubation at 30 °C with shaking at 200 rpm, optical density at 600 nm (OD_600_) was measured to assess growth. No significant inhibition was observed at concentrations up to 300 mM, with OD_600_ values remaining comparable to the non-saline control (0 mM: 1.20 ± 0.05; 100 mM: 1.15 ± 0.04; 200 mM: 1.10 ± 0.03; 300 mM: 1.00 ± 0.04). However, a marked decline occurred at extreme levels (>400 mM), with OD_600_ dropping to 0.60 ± 0.03 at 400 mM and 0.30 ± 0.02 at 500 mM.

### 2.2. Physicochemical Characterization of Biogenic Nano-Selenium

**Figure 3:**
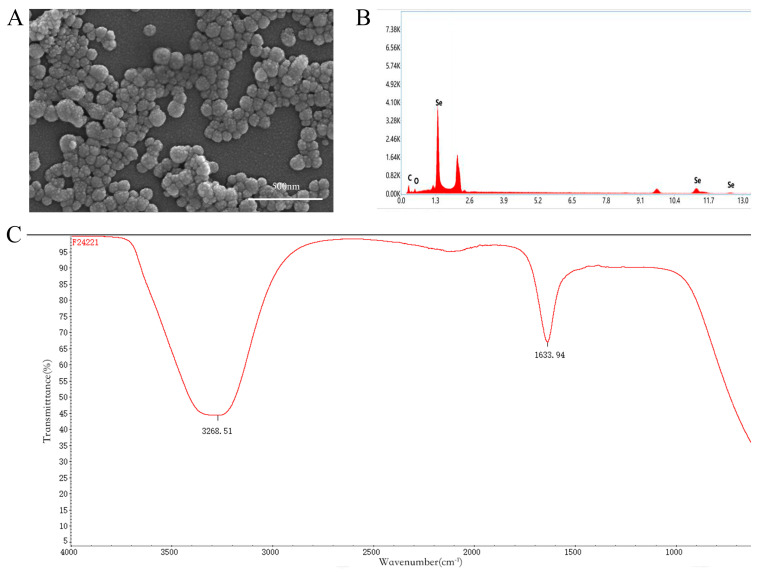
Physicochemical characteristics of SeNPs synthesized by *S. liquefaciens* SL-1. (**A**) SEM image; (**B**) EDS spectrum; (**C**) FTIR spectrum.

SEM imaging (Figure 3A) of SeNPs synthesized by strain SL-1 revealed large, irregular aggregates with coarse surfaces and nodular protrusions, suggesting involvement of extracellular polymers or biomolecular coatings during synthesis. Dispersed, spherical SeNPs with uniform morphology and diameters between 50 and 100 nm were also observed, indicating that primary particles formed initially and later aggregated under the influence of extracellular substances.

EDS analysis (Figure 3B) confirmed selenium as the major element (51.2% by mass), accompanied by C and O, indicating potential organic capping from bacterial metabolites. FTIR spectra (Figure 3C) revealed absorption peaks of hydroxyl (-OH ~3400 cm^−1^), carboxyl (C=O ~1650 cm^−1^), and amide I (N-H bending and C-N stretching ~1540 cm^−1^) functional groups, suggesting the presence of proteins and polysaccharides that likely enhance nanoparticle stability and biocompatibility.

### 2.3. Effects of Composite Inoculant on Rice Seedling Growth Under Saline–Alkali Stress

Under saline–alkali stress (CK2), rice seedling growth was significantly inhibited, with shoot height, fresh weight, and dry weight reduced by 37.1%, 48.9%, and 52.4%, respectively, compared to the control (CK1) (*p* < 0.05; Table 1). Application of SL-1 alone (S1) alleviated growth inhibition. Further significant improvements were observed in composite inoculant treatments (S2, S3, S4), with S3 (SL-1 + 40 mmol/L SeNPs) showing the best performance: increases of 31.6%, 53.8%, and 60.6% in plant height, shoot fresh weight, and shoot dry weight compared to CK2.

**Figure 4:**
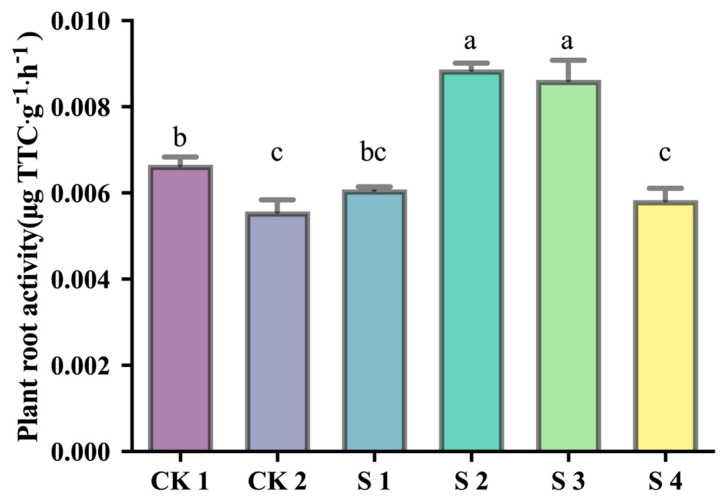
Effects of treatments on root activity under saline–alkali stress. Bars represent mean ± SD (*n* = 3). Different lowercase letters indicate significant differences (*p* < 0.05, Duncan’s test).

Root activity, a key indicator of stress resistance, was also significantly reduced under CK2. SL-1 (S1) improved root vigor, and composite inoculants (S2, S3) further enhanced it, with S2 showing the highest value (0.0087 μg TTC·g^−1^·h^−1^), a 59.2% increase over CK2 (*p* < 0.05). High SeNP concentration (S4) caused a decline compared to S3, suggesting potential inhibitory effects at excessive levels (Figure 4).

### 2.4. Effects of Compound Fungicide on Rice Yield and Selenium Content in Grains

Grain yield data at maturity (Table 2) showed that CK2 significantly reduced panicle weight and filled grain number, which were only 24.5% and 26.8% of CK1, respectively. S1 moderately restored yield parameters. All composite inoculant treatments (S2, S3, S4) significantly improved yield over CK2 and S1, with S3 showing the best results: panicle weight of 3.42 g (4.3-fold over CK2) and 115 filled grains (310.7% increase). Spikelet sterility was also reduced.

### 2.5. Effects of Compound Fungicides on Nutrient Availability in Rhizosphere Soil

Table 3 shows that CK2 soils had the lowest levels of TN, TP, TK, HN, AP, and AK. S1 significantly improved TN, TK, HN, and AP by 5.42%, 102.61%, 11.86%, and 17.78%, respectively. TP and AK increased slightly but not significantly. Composite inoculants (S2–S4) further improved soil nutrients. Notably, S3 enhanced TK (316.52%), HN (+26.10%), and AP (+23.70%) most effectively. S4 significantly raised TN (+9.83%), while S2 significantly raised AK (+9.37%).

### 2.6. Selenium Accumulation and Translocation in Rice

Selenium was predominantly accumulated in roots, followed by stems, leaves, and grains (Table 4), indicating root-based retention and limited upward translocation. Increasing SeNP doses led to higher Se in all tissues. High Se enrichment in roots and stems under S4 suggests organ-specific distribution regulated by dose and transport efficiency.

Grain selenium content increased dose-dependently with SeNP concentration. S2 and S3 yielded 205.0 and 234.1 μg/kg, respectively, meeting the Chinese national standard for Se-enriched rice (40–300 μg/kg). S4 reached 628.8 μg/kg, exceeding safe limits.

### 2.7. Effects of Compound Fungicides on Rhizosphere Microbial Community Structure

**Figure 5:**
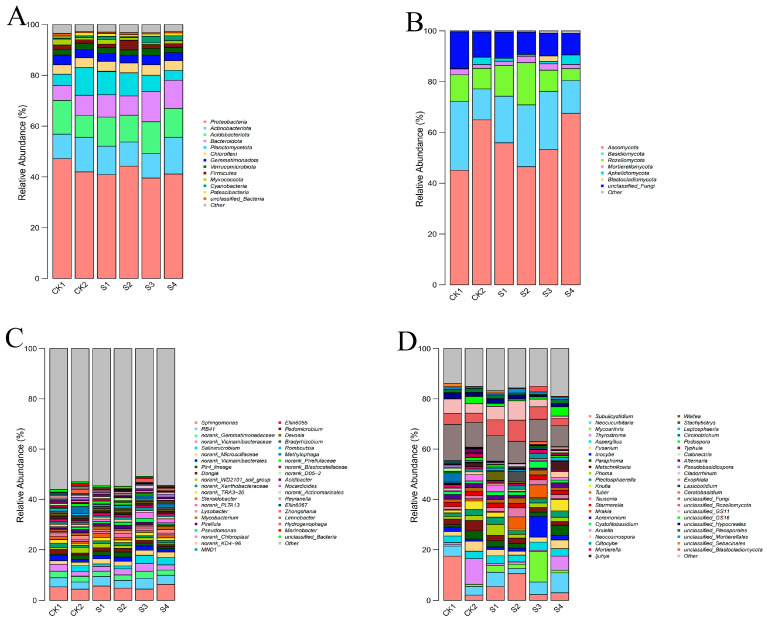
Relative abundance of dominant microbial phyla and genera in rhizosphere soil under different treatment conditions. (**A**) Bacterial phylum; (**B**) Fungi phylum; (**C**) Bacterial genus; (**D**) Fungi genus.

Illumina NovaSeq sequencing of 16S rRNA (bacteria) and ITS1 (fungi) regions revealed changes in microbial community composition and diversity. Bacterial communities were dominated by Proteobacteria, Actinobacteriota, and Acidobacteriota (Figure 5A). CK2 had reduced Proteobacteria, while S2 and S3 restored their abundance, especially in S2 (>50%). Fungal communities (Figure 5B) were dominated by Ascomycota and Basidiomycota, with S3 showing significant enrichment of Ascomycota. The bacterial community analysis at the genus level was dominated by *Sphingomonas*, *RB41*, and *norank_Gemmatimonadaceae* (Figure 5C). The CK2 strain showed reduced Sphingomonas abundance, while Sphingomonas increased in groups S1 and S4. Fungal communities (Figure 5D) were primarily composed of *Subulicystidium*, *Neocucurbitaria*, and *Mycoarthris*, with group S2 showing significant enrichment of *Subulicystidium*.

**Figure 6:**
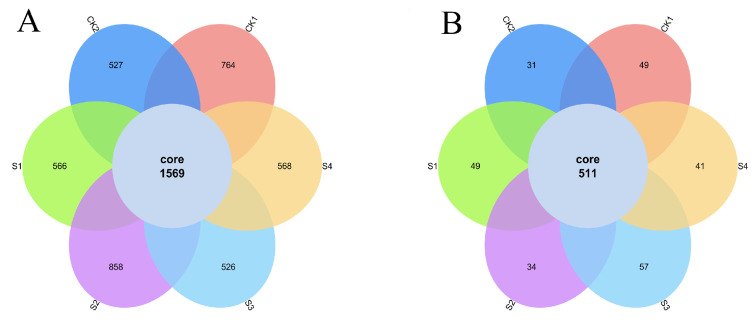
Venn diagrams showing unique and shared OTUs among treatments. (**A**) Bacterial OTUs; (**B**) Fungal OTUs.

Venn diagram analysis (Figure 6) indicated increased OTU richness with composite inoculants. S2 had the most unique bacterial OTUs (40), while S3 had the most unique fungal OTUs (14), highlighting SeNPs concentration-dependent specificity.

**Figure 7:**
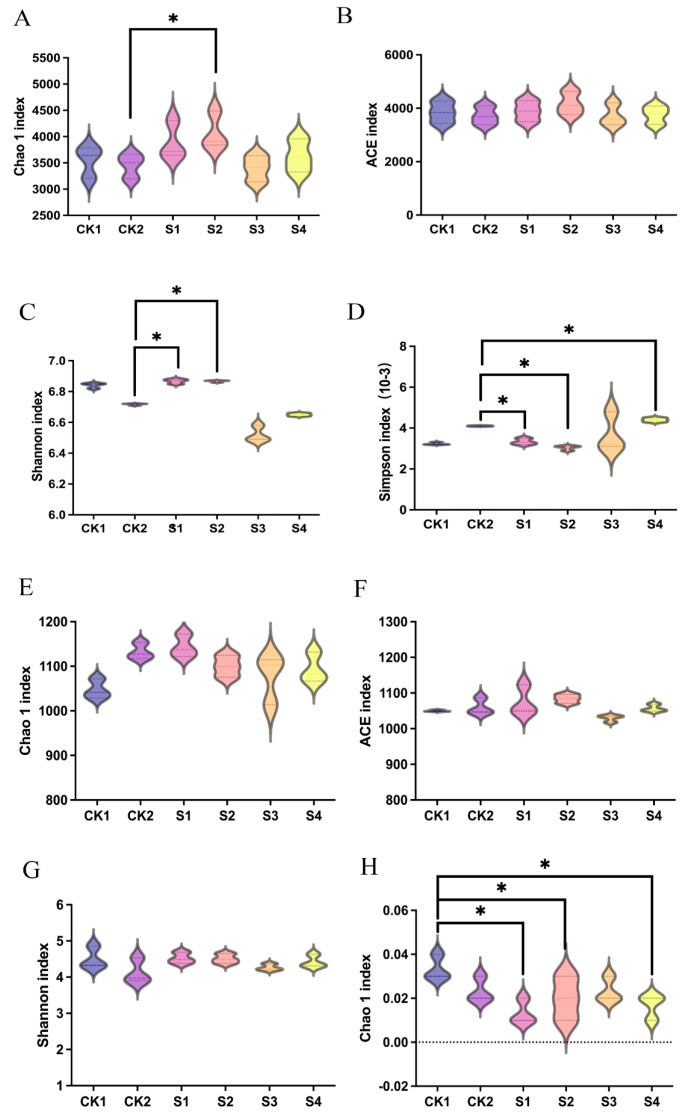
Alpha diversity indices of microbial communities under different treatments. (**A**–**D**) Bacterial indices; (**E**–**H**) Fungal indices. * Indicates significant difference (*p* < 0.05, Duncan’s test).

Alpha diversity indices were significantly improved by composite treatments. S2 increased bacterial Chao1 and Shannon indices while decreasing the Simpson index (Figure 7A–D). S3 notably enhanced the fungal Shannon index and decreased the Simpson index (Figure 7E,F), suggesting more stable, diverse fungal communities.

In summary, composite inoculants—particularly S2 for bacteria and S3 for fungi—effectively restored microbial community structure and diversity under saline–alkali stress, supporting a more resilient rhizosphere ecosystem.

## 3. Discussion

### 3.1. Alleviation of Saline–Alkali Stress by ACC Deaminase Activity of Serratia liquefaciens

In this study, a highly active ACC deaminase-producing strain of *Serratia liquefaciens* (SL-1) was successfully isolated from the rice rhizosphere. ACC deaminase breaks down ACC, the direct precursor of ethylene, thereby lowering ethylene levels in plants under stress conditions such as saline–alkali stress [5,6,7]. Ethylene, while an essential phytohormone, inhibits root elongation and overall development when overaccumulated [2,3]. Application of SL-1 (S1 treatment) significantly improved rice growth and root activity under stress (Table 1), in line with the ethylene attenuation mechanism proposed by Glick et al. [5,6,7]. Additionally, as a PGPR, *S. liquefaciens* may promote growth through multiple mechanisms such as auxin production, phosphate solubilization, nitrogen fixation, or siderophore production [10,24], collectively enhancing plant stress tolerance.

The newly conducted salinity tolerance tests demonstrate that *Serratia liquefaciens* SL-1 possesses moderate halotolerance, enabling it to withstand salt concentrations up to 300 mM NaCl/Na_2_CO_3_ without significant growth impairment. This level aligns with the saline–alkali stress conditions in our pot experiments (EC ~5.6 dS/m), suggesting effective rhizosphere colonization and survival in affected soils. The minimal impact on growth at relevant concentrations ensures that key PGP traits, such as ACC deaminase activity, remain functional, thereby sustaining ethylene modulation and stress alleviation in rice plants [5,6,7]. Furthermore, the unchanged efficiency of nano-selenium biosynthesis under these conditions (Section 2.1) indicates that SeNP production and stability are preserved, avoiding potential disruptions to the composite inoculant’s synergistic effects. This halotolerance is consistent with reports on other PGPR strains isolated from saline environments, where adaptive mechanisms like osmolyte accumulation and ion exclusion maintain metabolic activity [8,9,10]. Overall, these findings validate SL-1′s suitability for biofertilizer development in saline–alkali regions, enhancing its potential to improve plant growth, nutrient uptake, and Se biofortification without confounding environmental factors.

### 3.2. Characteristics of Biogenic Nano-Selenium and Its Synergistic Effect with S. liquefaciens

Biogenic SeNPs synthesized by SL-1 were within the 50–100 nm range (Figure 2A). FTIR analysis confirmed the presence of hydroxyl, carboxyl, and amide functional groups on the nanoparticle surface—organic capping primarily derived from bacterial metabolites such as extracellular polysaccharides and proteins. This bio-coating enhances aqueous dispersibility and stability, preventing rapid aggregation [16,25], and may also improve biocompatibility and uptake efficiency in plants [15,26].

Importantly, the combination of *S. liquefaciens* and SeNPs (e.g., S3 treatment) significantly outperformed SL-1 alone (S1) in alleviating salt stress, enhancing growth, and increasing yield (Table 1 and Table 2), consistent with findings by Alharbi et al. [27]. This synergy may be attributed to the following: (1) SL-1 improving plant physiological status and enabling better selenium uptake; (2) SeNPs exhibiting antioxidant and regulatory roles at optimal concentrations; and (3) joint modulation of antioxidant systems, osmotic adjustment, and ionic homeostasis by PGPR and SeNPs.

The observed synergy between SL-1 and SeNPs can be attributed to multifaceted mechanisms that enhance plant resilience under saline–alkali stress. Firstly, SL-1′s ACC deaminase activity modulates ethylene levels, reducing stress-induced hormonal imbalances, while SeNPs contribute to antioxidant defense by scavenging reactive oxygen species (ROS) and improving osmotic adjustment [15,27]. This combined action likely amplifies ionic homeostasis and nutrient uptake, as evidenced by the significant increases in biomass and yield in the S3 treatment (Table 1 and Table 2). Furthermore, recent studies on nanoparticle-PGPR interactions under salinity, such as those involving zinc oxide nanoparticles and halotolerant Bacillus strains, demonstrate enhanced microbial colonization and plant physiological responses, including elevated antioxidant enzyme activity and reduced Na^+^ accumulation. These findings align with our results, where the strain’s confirmed salinity tolerance (Section 3.1) ensures stable PGP trait expression, mitigating potential confounding effects from environmental stress. Overall, the composite inoculant’s consistency in promoting growth without toxicity at optimal doses (20–40 mmol/L) underscores its potential for field applications.

### 3.3. Composite Inoculant Enables Safe and Effective Selenium Biofortification in Rice

Selenium is essential for human health, and biofortification of staple crops is a promising strategy to improve dietary Se intake [28,29]. The results demonstrate that composite inoculants with 20–40 mmol/L SeNPs (S2 and S3) significantly increased grain Se content to 205.0–234.1 μg/kg, meeting national Se-enriched rice standards without adverse effects on plant growth (Table 4). This validates the feasibility of PGPR-SeNPs-based biofortification.

However, the S4 treatment (80 mmol/L SeNPs) led to excessively high Se levels in grains (628.8 μg/kg), exceeding safe limits and diminishing growth/yield benefits (Table 1 and Table 2), indicating potential toxicity at high concentrations. Tissue-specific Se accumulation followed the pattern: root > stem > leaf > grain (Table 4), consistent with the literature suggesting root retention and limited xylem transport of selenium [30].

### 3.4. Positive Regulation of Soil Nutrients and Microbial Communities by the Composite Inoculant

The ACC deaminase-based PGPR and nano-selenium composite not only mitigated stress-induced growth suppression but also significantly improved rhizosphere nutrient profiles and microbial community structure.

Nutrient enhancement was evident in all key elements (N, P, K) under stress (Table 3). Notably, TK in S3 (4.79 g/kg) was 4.2 times higher than CK2. HN and AP were also significantly elevated by 26.1% and 23.7%, respectively.

Moreover, increased microbial diversity and functional redundancy underpin ecological resilience. S2 significantly enhanced bacterial diversity, while S3 improved fungal diversity (Figure 7), increasing the system’s ecological redundancy—wherein functionally similar taxa can substitute under disturbance, enhancing system stability. This ecological buffering is vital under saline–alkali conditions.

Enrichment of functional taxa was also observed. Proteobacteria and Actinobacteriota include nitrogen-fixers, cellulolytic bacteria, and IAA producers; Ascomycota and Basidiomycota contribute to phosphorus cycling, humus formation, and pathogen suppression. These shifts support nutrient turnover and facilitate stable plant–microbe networks.

Notably, a dose-dependent effect was observed: S2 (20 mmol/L) was most beneficial for bacteria, while S3 (40 mmol/L) optimized fungal communities. In contrast, S4 caused partial declines, suggesting high SeNP concentrations may disrupt microbial balance. Hence, dosage optimization is crucial for ecological safety.

In summary, the composite inoculant optimized the rhizosphere ecosystem through dual pathways: enhancing nutrient accessibility and cycling and increasing microbial diversity and redundancy. This strategy offers a robust framework for sustainable, stress-resilient biofertilizer development.

### 3.5. Limitations

While the findings of this study are encouraging, several limitations remain. All experiments were conducted in pot-based greenhouse conditions, which may not fully reflect field-scale variability and environmental complexity. The environmental behavior and ecological fate of nano-selenium—especially its long-term persistence, mobility in soil, and potential effects on non-target organisms—are still not well understood. Additionally, important agronomic parameters such as the optimal application rate, timing, and delivery methods of the composite bioinoculant require further refinement to enhance efficacy and safety. Finally, the generalizability of this approach across different rice varieties and other crop species warrants systematic exploration to support broader agricultural application.

## 4. Materials and Methods

### 4.1. Plant Materials and Soil Preparation

The rice (*Oryza sativa* L.) cultivar ‘Zhongfa No. 5′ was used in this study. Soil was collected from the farmland of the West Campus of Changchun Normal University (0–20 cm depth; 125°23.74′ E, 43°54.83′ N), air-dried, cleaned of debris, and sieved through a 2 mm mesh.

To simulate saline–alkali stress, part of the soil was amended with analytical-grade NaCl and Na_2_CO_3_ (1:1, *w*/*w*), adjusting the electrical conductivity (EC) of a 1:5 soil–water extract to approximately 5.6 dS/m. This served as the stress treatment soil. The unamended soil was used as the control. All treated soils were stored in the dark at 4 °C prior to use.

### 4.2. Screening and Identification of ACC Deaminase-Producing Bacteria

#### 4.2.1. Enrichment and Isolation of Bacterial Strains

Rhizosphere soil (10 g) was suspended in sterile saline and serially diluted. Aliquots were plated on PAF enrichment medium (peptone 10 g, casein hydrolysate 10 g, MgSO_4_·7H_2_O 1.5 g, K_2_HPO_4_ 1.5 g, glycerol 10 mL) and incubated at 28 °C for 2–3 days. Single colonies were then transferred to ADF medium, where ACC served as the sole nitrogen source, for functional screening. The composition of ADF agar included Na_2_HPO_4_ (6.0 g), KH_2_PO_4_ (4.0 g), MgSO_4_·7H_2_O (0.2 g), glucose (2.0 g), gluconic acid (2.0 g), citric acid (2.0 g), FeSO_4_·7H_2_O (1 mg), H_3_BO_3_ (0.01 mg), MnO_3_ (0.01 mg), ZnSO_4_·7H_2_O (0.1246 mg), CuSO_4_·5H_2_O (0.0782 mg), and ACC (5 mmol/L), adjusted to pH 7.2.

Purified isolates were preserved at −80 °C in LB broth supplemented with 20% (*v*/*v*) glycerol.

#### 4.2.2. Determination of ACC Deaminase Activity

ACC deaminase activity was assayed with slight modifications based on Honma and Shimomura (1978) [31] and Penrose and Glick (2003) [32]. Briefly, activated strains were cultured in ADF liquid medium containing ACC. Cells were harvested and washed with 0.1 M Tris-HCl buffer (pH 8.5), resuspended, and lysed with 2% (*v*/*v*) toluene. The bacterial suspension was then incubated with 0.5 M ACC solution. After reaction termination with 0.56 M HCl, the supernatant was reacted with 2,4-dinitrophenylhydrazine (DNPH) reagent, and absorbance was measured at 540 nm. The α-ketobutyrate concentration was determined using a standard curve. Protein content was quantified using the Bradford assay. One unit (U) of enzyme activity was defined as the amount of enzyme that produced 1 μmol of α-ketobutyrate per minute per mg of protein (μmol protein^−1^·min^−1^).

#### 4.2.3. Molecular Identification of the Isolate

The strain with the highest ACC deaminase activity was selected for molecular identification. Genomic DNA was extracted using a bacterial genomic DNA kit (DP302, Tiangen Biotech, Beijing, China). The 16S rRNA gene was amplified using universal primers 27F and 1492R. The PCR system (50 μL) was prepared using 2× AceTaq Master Mix (P412, Vazyme, Nanjing, China), with the following program: 95 °C for 5 min; 40 cycles of 95 °C for 15 s, 56 °C for 20 s, and 72 °C for 40 s; and a final extension at 72 °C for 10 min.

PCR products were confirmed by agarose gel electrophoresis and sent for commercial sequencing (Sangon Biotech, Shanghai, China). Sequences were analyzed by BLAST in NCBI, and a phylogenetic tree was constructed using MEGA 11 with bootstrap analysis.

### 4.3. Biosynthesis and Characterization of Nano-Selenium

#### 4.3.1. Biosynthesis of SeNPs

The selected isolate (designated SL-1) was inoculated into LB broth (2% *v*/*v*) and cultured at 30 °C, 200 rpm, until mid-log phase (OD_600_ ≈ 0.8). Sodium selenite (Na_2_SeO_3_) was added to a final concentration of 5 mmol/L, followed by 48 h of dark incubation. The formation of SeNPs was indicated by a color change from pale yellow to brick red.

The culture was centrifuged at 10,000× *g*, 4 °C for 15 min to collect red precipitates. The SeNPs were washed 3–5 times with sterile deionized water and freeze-dried. The final powder was stored at 4 °C.

#### 4.3.2. Physicochemical Characterization of SeNPs

Morphology and Elemental Composition: Field-emission scanning electron microscopy (FESEM, Zeiss Gemini SEM 500 (Zeiss, Oberkochen, Germany), 10.0 kV) was used to observe particle morphology, and energy-dispersive X-ray spectroscopy (EDS, Oxford Instruments, Oxford, UK) was used for elemental analysis.

Particle Size and Zeta Potential: Dynamic light scattering (DLS) was performed using a Malvern Zetasizer Nano ZS (Malvern Panalytical, Almelo, The Netherlands) to measure hydrodynamic diameter and zeta potential of the SeNPs in aqueous suspension.

Surface Functional Groups: Fourier-transform infrared spectroscopy (FTIR, Bruker Tensor 27, Bruker, Billerica, MA, USA) was conducted over the range of 4000–400 cm^−1^ to identify functional groups on the nanoparticle surface.

### 4.4. Pot Experiment and Bioformulation

#### 4.4.1. Preparation of Composite Bioinoculant

SL-1 cells were harvested at late log phase (OD_600_ ≈ 0.8), washed, and resuspended in sterile saline to ~2 × 10^8^ CFU/mL. SeNPs powder was ultrasonically dispersed (100 W, 2 min) in sterile deionized water to prepare 20, 40, and 80 mmol/L suspensions. Composite inoculants were freshly prepared by mixing equal volumes of bacterial suspension and SeNPs solution prior to application.

#### 4.4.2. Pot Trial Design

The pot experiment was conducted in a controlled greenhouse at Changchun Normal University (light intensity: 3000 Lux; temperature: 25 °C; humidity: 70%). A completely randomized block design was used with six treatments and three replicates per treatment:

CK1: Non-saline soil + sterile water;

CK2: Saline–alkali soil + sterile water;

S1: Saline–alkali soil + SL-1 suspension;

S2: Saline–alkali soil + SL-1 + 20 mmol/L SeNPs;

S3: Saline–alkali soil + SL-1 + 40 mmol/L SeNPs;

S4: Saline–alkali soil + SL-1 + 80 mmol/L SeNPs.

Each pot was filled with 2 kg of soil. Surface-sterilized (1% NaClO) and pre-germinated seeds of ‘Zhongfa No. 5′ were transplanted into each pot (five seedlings per pot). Inoculations were applied on days 7 and 14 after transplantation with 50 mL of the respective treatments. CK1 and CK2 received sterile water. No fertilizers were added, and soil moisture was maintained at 70–80% field capacity.

### 4.5. Measurement Parameters and Methods

#### 4.5.1. Rice Growth and Yield Measurements

At 21 days post-treatment, plant height and fresh/dry weight of aboveground parts were recorded. Root activity was determined using the TTC reduction method [33]. At maturity (90 days), the following parameters were assessed: number of effective panicles per plant, total grains per panicle, filled grains per panicle, seed-setting rate, 1000-grain weight, and grain yield per plant. The primary focus was on panicle weight, number of filled grains per panicle, and empty spikelet rate.

#### 4.5.2. Selenium Content in Plant Tissues

At maturity, plant tissues (roots, stems, leaves, and grains) were collected, washed (including 0.1 mol/L HCl treatment), dried, and ground. Samples were digested with HNO_3_-HClO_4_, and Se content was measured using atomic fluorescence spectrometry (AFS-3100, Beijing Haiguang Instrument Co., Ltd., Beijing, China) [34]. Results are expressed as μg Se per kg dry weight.

#### 4.5.3. Soil Physicochemical Analysis

On day 21, rhizosphere soil was sampled for nutrient analysis following Chinese national standard GB/T 22923-2008 [35]:

Total nitrogen (TN): semi-micro Kjeldahl method [36];

Total phosphorus (TP): NaOH fusion–molybdenum antimony colorimetry [37];

Total potassium (TK): NaOH fusion–flame photometry [38];

Available nitrogen (AN): alkaline hydrolysis diffusion method [36];

Available phosphorus (AP): 0.5 mol/L NaHCO_3_ extraction–colorimetry [39];

Available potassium (AK): 1 mol/L NH_4_OAc extraction–flame photometry [40].

#### 4.5.4. Rhizosphere Microbial Community Analysis

On day 21, rhizosphere soils were collected (pull out the roots, pat the roots to remove loose soil clumps, and use sterilized brushes to collect soil samples that cling to the surface of the roots) and stored at −80 °C. Soil DNA was extracted using the TIANamp Soil DNA Kit.

The bacterial 16S rRNA V3–V4 region (primers 341F/805R) and fungal ITS1 region (primers ITS1F/ITS2) were amplified and sequenced (Sangon Biotech, Shanghai, China; Illumina NovaSeq PE250; Illumina, San Diego, CA, USA). Raw reads were quality-filtered, merged, and chimera-checked. Operational taxonomic units (OTUs, 97% similarity) or amplicon sequence variants (ASVs) were obtained using QIIME2.

Alpha diversity indices (Chao1, ACE, Shannon, and Simpson) were calculated. Taxonomic annotation was based on the Silva (bacteria) and UNITE (fungi) databases. Statistical analyses and visualizations were conducted using R (vegan and phyloseq packages, 4.3.2).

### 4.6. Statistical Analysis

All data were analyzed using one-way ANOVA in SPSS 24.0, followed by Duncan’s multiple range test (*p* < 0.05). Graphs were generated using GraphPad Prism 10. Results are expressed as mean ± standard deviation (mean ± SD).

## 5. Conclusions

This study developed a composite bioinoculant combining the ACC deaminase-producing *Serratia liquefaciens* SL-1 with biosynthesized nano-selenium (SeNPs) and evaluated its impact on rice under saline–alkali stress. SL-1, isolated from the rice rhizosphere, exhibited high ACC deaminase activity (0.084 U/mg protein), contributing to ethylene regulation and enhanced stress tolerance. The SeNPs synthesized by SL-1 had uniform particle sizes (80–120 nm) and were capped with organic functional groups, enhancing their stability and potential bioavailability.

Under saline–alkali conditions, the composite inoculant—particularly the formulation containing 40 mmol/L SeNPs—significantly improved rice seedling biomass, root vigor, panicle weight, and seed-setting rate, exceeding the effects of PGPR treatment alone. Grain selenium concentrations in treatments with 20–40 mmol/L SeNPs reached 205.0–234.1 μg/kg, aligning with national standards for selenium-enriched rice. Importantly, this was achieved without negative impacts on plant growth, unlike the excessive selenium accumulation observed with 80 mmol/L SeNPs.

In addition to growth promotion and nutritional enhancement, the composite inoculant improved rhizosphere soil fertility, increasing the contents of total potassium, available nitrogen, and available phosphorus. It also reshaped the microbial community structure, enhancing alpha diversity and functional redundancy. Bacterial and fungal communities responded differently to SeNP concentrations, suggesting a dose-dependent modulation of microbial dynamics.

Overall, the combined application of ACC deaminase-producing PGPR and biogenic nano-selenium presents a promising, eco-friendly approach to mitigating saline–alkali stress, enhancing soil health, improving crop yield and nutritional quality, and fostering beneficial rhizosphere interactions. This strategy offers a scientifically grounded and practically viable direction for the development of sustainable biofertilizers.

## Figures and Tables

**Table 1:** Effects of treatments on rice seedling growth under saline–alkali stress.

Treatment	Shoot Fresh Weight (g/plant)	Shoot Dry Weight (g/plant)	Plant Height (cm)
CK1	0.22 ± 0.02 a	0.04 ± 0.00 ab	12.53 ± 0.52 ab
CK2	0.11 ± 0.01 c	0.02 ± 0.01 c	9.93 ± 0.15 e
S1	0.14 ± 0.01 b	0.03 ± 0.00 b	11.50 ± 0.30 cd
S2	0.18 ± 0.02 b	0.04 ± 0.00 b	12.07 ± 0.32 bc
S3	0.23 ± 0.03 a	0.05 ± 0.01 a	13.07 ± 0.57 a
S4	0.16 ± 0.01 b	0.04 ± 0.00 b	10.80 ± 0.62 d

Note: Values are mean ± SD (*n* = 3). Different lowercase letters indicate significant differences (*p* < 0.05, Duncan’s test).

**Table 2:** Effects of treatments on rice yield components under saline–alkali stress.

Treatment	Panicle Weight (g)	Filled Grains/Panicle	Spikelet Sterility (%)
CK1	2.45 ± 0.73 b	85 ± 18.25 b	3.18
CK2	0.79 ± 0.18 d	28 ± 7.08 d	4.21
S1	1.42 ± 0.35 c	48 ± 15.06 c	2.43
S2	2.48 ± 0.80 b	83 ± 21.14 b	3.07
S3	3.42 ± 0.34 a	115 ± 13.82 a	2.93
S4	1.09 ± 0.37 cd	39 ± 15.76 cd	1.53

Different lowercase letters indicate significant differences (*p* < 0.05, Duncan’s test).

**Table 3:** Effects of treatments on rhizosphere soil nutrient contents under saline–alkali stress.

Treatment	TN (g/kg)	TP (g/kg)	TK (g/kg)	HN (mg/kg)	AP (mg/kg)	AK (mg/kg)
CK1	3.05 ± 0.06 bc	0.95 ± 0.06 a	4.21 ± 0.03 c	279.90 ± 2.02 a	30.40 ± 0.38 d	78.17 ± 2.75 ab
CK2	2.95 ± 0.06 c	0.81 ± 0.08 b	1.15 ± 0.04 f	209.58 ± 0.62 f	27.00 ± 0.37 e	74.07 ± 4.25 b
S1	3.11 ± 0.04 b	0.85 ± 0.05 ab	2.33 ± 0.04 d	234.44 ± 0.75 e	31.80 ± 1.02 c	79.03 ± 2.66 ab
S2	3.07 ± 0.10 bc	0.86 ± 0.03 ab	2.04 ± 0.04 e	259.41 ± 1.55 c	36.20 ± 0.71 a	81.01 ± 2.64 a
S3	3.15 ± 0.04 ab	0.87 ± 0.04 ab	4.79 ± 0.07 a	264.29 ± 0.33 b	33.40 ± 0.69 b	75.02 ± 2.00 b
S4	3.24 ± 0.09 a	0.79 ± 0.04 b	4.68 ± 0.05 b	244.68 ± 1.37 d	32.00 ± 0.48 c	75.00 ± 1.00 b

Different lowercase letters indicate significant differences (*p* < 0.05, Duncan’s test).

**Table 4:** Selenium content (μg/kg dry weight) in different rice tissues under various treatments.

Treatment	Root	Stem	Leaf	Grain
CK1	0.35 ± 0.06	0.03 ± 0.00	0.00 ± 0.00	0.00 ± 0.00
CK2	0.00 ± 0.00	0.00 ± 0.00	0.00 ± 0.00	0.00 ± 0.00
S1	0.00 ± 0.00	0.00 ± 0.00	0.00 ± 0.00	0.00 ± 0.00
S2	988.76 ± 29.14	552.93 ± 10.22	394.77 ± 9.37	205.04 ± 9.31
S3	1636.28 ± 35.85	908.02 ± 13.57	391.81 ± 8.78	234.13 ± 6.61
S4	3319.26 ± 8.52	1806.10 ± 17.38	996.18 ± 12.02	628.79 ± 8.80

## Data Availability

The data that support the findings of this study are available from the corresponding author upon reasonable request.
